# Pingchan Granule for Motor Symptoms and Non-Motor Symptoms of Parkinson’s Disease: A Randomized, Double-Blind, Placebo-Controlled Study

**DOI:** 10.3389/fphar.2022.739194

**Published:** 2022-02-25

**Authors:** Si-Chun Gu, Qing Ye, Chang-De Wang, Shao-Rong Zhao, Jie Zhou, Chen Gao, Yu Zhang, Zhen-Guo Liu, Can-Xing Yuan

**Affiliations:** ^1^ Department of Neurology, Longhua Hospital, Shanghai University of Traditional Chinese Medicine, Shanghai, China; ^2^ Department of Neurology, Shanghai TCM-integrated Hospital, Shanghai University of Traditional Chinese Medicine, Shanghai, China; ^3^ Department of Neurology, Putuo District Central Hospital, Shanghai University of Traditional Chinese Medicine, Shanghai, China; ^4^ Department of Neurology, Xinhua Hospital, Shanghai Jiao Tong University School of Medicine, Shangha, China

**Keywords:** pingchan granule, Parkinson’s disease, traditional Chinese medicine, motor function, non-motor function

## Abstract

**Background:** Pingchan granule (PCG) is a traditional Chinese medicine for treating Parkinson’s disease (PD).

**Objective:** This study aimed at evaluating the efficacy and safety of PCG for motor and non-motor symptoms of PD.

**Methods:** In this multicenter, randomized, double-blind, placebo-controlled trial, 292 participants with mild-to-moderate PD were included and followed for 36 weeks (24 week treatment, 12-week follow-up after intervention), randomly assigned at a 1:1 ratio to receive PCG or placebo. The primary outcomes included the severity of motor symptoms assessed by the Unified Parkinson’s disease Rating Scale (UPDRS) part 3 (UPDRS-III) score and the rate of disease progression assessed by the total UPDRS score. Secondary outcomes included non-motor symptoms assessed using the Scale for Outcomes in Parkinson’s Disease-Autonomic (SCOPA-AUT), Parkinson’s disease Sleep Scale (PDSS), 24-item Hamilton Rating Scale for Depression (HAM-D), Hamilton Rating Scale for Anxiety (HAM-A), UPDRS part 2 (UPDRS-II), and 39-item Parkinson’s Disease Questionnaire (PDQ-39) scores. Assessments were done at baseline (T0), 12 weeks (T1), 24 weeks (T2), and 36 weeks (T3).

**Results:** Generalized estimating equation analyses revealed that the PCG group had significantly better improvement in UPDRS-III score at T1, T2, and T3 [time-by-group interaction, T1: β, −0.92 (95% CI, −1.59–−0.25; *p* = 0.01); T2: β, −2.08 (95% CI, −2.90–−1.27; *p* < 0.001); T3: β, −4.54 (95% CI, −5.37–−3.71; *p* < 0.001))]. The PCG group showed a greater decrease (rate of disease change) in the total UPDRS score between T0 and T2 [−2.23 (95% CI, −2.72–−1.73; *p* < 0.001) points per week vs*.* −0.21 (95% CI, −0.80–0.39; *p* = 0.50) points per week in the placebo group, *p* < 0.001]. Ameliorations of SCOPA-AUT, PDSS, HAM-D, HAM-A, UPDRS-II, and PDQ-39 scores were also observed.

**Conclusion:** PCG had a long-lasting and extensive symptomatic efficacy for both motor and non-motor symptoms of PD with good tolerance.

**Trial registration:** Chinese Clinical Trial Register, ChiCTR-INR-17011949.

## Background

It is increasingly recognized that Parkinson’s disease (PD) is a common neurodegenerative disease with heterogeneous symptomatology, characterized by the inexorable progression of motor symptoms and a wide range of non-motor symptoms including sleep disturbances, autonomic dysfunction, neuropsychiatric disorders, and cognitive impairment (Armstrong and Okun, 2020). The combination of these motor and non-motor symptoms can cause severe disability in patients and impose heavy burdens for their caregivers (Schapira et al., 2017). Dopamine replacement therapy and subthalamic deep-brain stimulation (DBS) are established treatments for PD; unfortunately, some motor and non-motor symptoms in PD do not seem to respond well to levodopa, DBS, or other forms of dopaminergic medications (such as monoamine oxidase-B [MAO-B] inhibitors and dopamine agonists) or appear to be resistant to such dopaminergic treatments with increased PD duration and disease progression. Furthermore, multi-neurotransmitter dysfunction involving not just the dopaminergic pathways but also serotonergic, noradrenergic, and cholinergic pathways in the brain might underlie the large range of non-motor symptoms of PD. Due to the multiple causative factors involved, challenges persistently exist in maintaining extensive and sufficient symptomatic both motor and non-motor control for PD patients. However, there is little evidence showing that these patients would benefit substantially from one particular class of anti-parkinsonian medications in terms of both motor and prevailing non-motor symptoms simultaneously (Chaudhuri et al., 2010).

In China, Pingchan granule (PCG), as a traditional Chinese medicine (TCM), was summed up based on the empirical clinical practice of a TCM expert Jian-Hua Hu and has been widely used in PD treatment for decades, showing very little toxicity or side effects, complementary to the existing anti-parkinsonian pharmacotherapy and functional surgery. PCG (produced by Jiangyin Tianjiang Pharmaceutical Co., Ltd., Jiangyin, China; batch number: 1410302) consists of 6 commonly used Chinese herbs: Lycium barbarum L., 12 g; Taxillus chinensis (DC.) Danser, 15 g; Gastrodia elata Blume, 9 g; Paeonia lactiflora Pall., 15 g; Arisaema erubescens (Wall.) Schott, 15 g; and Curcuma phaeocaulis Valeton, 9 g, and 3 commonly used traditional Chinese medicinal materials: *Bombyx mori* Linnaeus, 9 g; Buthus martensii Karsch, 3 g; and Scolopendra subspinipes mutilans L. Koch, 3 g (Table 1). All taxonomic names of 6 plant and 3 non-plant medicinal materials have been verified by use of the Pharmacopoeia of China (2015) or http://www.theplantlist.org/. The standard analytic method of ultrahigh-performance liquid chromatography-mass spectrometry (UPLC-Q-TOF/MS) was used to examine the composition of the TCM formula granules, and eight compounds were identified from PCG through database comparison and references. Moreover, the results of mass spectrometry analysis of PCG and its specific ingredients were contained in the supplementary materials (see Supplementary Material for details).

**TABLE 1:** Scientific name, pharmaceutical name, parts and form used, and Chinese name of the corresponding components in Pingchan granule with its voucher number.

Scientific name	Pharmaceutical name	Parts and form used	Chinese name	Voucher no.
Lycium barbarum L.	Lycii fructus	Dried ripe fruit	Gou Qi Zi	PCG20180101
Taxillus chinensis (DC.) Danser	Taxilli herba	Dried branch, with leaf	Sang Ji Sheng	PCG20180102
Gastrodia elata Blume.	Gastrodiae rhizoma	Dried tuber	Tian Ma	PCG20180103
Paeonia lactiflora Pall.	Paeoniae alba radix	Dried root	Shao Yao	PCG20180104
Arisaema erubescens (Wall.) Schott.	Arisaematis rhizoma	Dried tuber	Tian Nan Xing	PCG20180105
Curcuma phaeocaulis Valeton.	Curcumae radix	Dried root tuber	E Shu	PCG20180106
*Bombyx mori* Linnaeus.	*Bombyx batryticatus*	Dried larva	Jiang Can	PCG20180107
Buthus martensii Karsch	Scorpio	Dried imago	Quan Xie	PCG20180108
Scolopendra subspinipes mutilans L. Koch	Scolopendra	Dried imago	Wu Gong	PCG20180109

In previous clinical trials, PCG did not only provide beneficial effects for motor symptoms including bradykinesia, tremor, rigidity, and motor complications such as dyskinesia and wearing-off but also show treatment efficacy in non-motor symptoms including autonomic impairment, depression, anxiety, and cognitive decline (Ye et al., 2014a; Ye et al., 2016a; Ye et al., 2018). However, the cited literature was limited by the relatively small number of subjects. The absence of strong evidence-based research might be the main obstacle toward the globalization of PCG.

It has been identified that dopaminergic cell loss might be nonlinear in the disease course, initially decreasing exponentially and slowing thereafter with advancing PD severity. This theory predicts that PD patients tend to show a rapid clinical progression at the early and middle stages of the disease, characterized by the deterioration of motor and non-motor symptoms, which highlights the need for adequate interventions (Hilker et al., 2005; Rascol et al., 2011). In order to further verify the clinical efficacy and safety of PCG at the non-advanced stage of PD, we conducted this randomized controlled trial with more participants and standardized outcome measures, and a longer follow-up period, and targeted therapy in patients with PD.

## Materials and Methods

### Study Design

This multicenter, randomized, double-blind, placebo-controlled trial was conducted in the neurology departments from 4 university hospitals in China (Longhua Hospital Affiliated to Shanghai University of TCM, Xinhua Hospital Affiliated to Shanghai Jiao Tong University School of Medicine, Shanghai TCM-integrated Hospital Affiliated to Shanghai University of TCM, and Putuo District Central Hospital Affiliated to Shanghai University of TCM). This clinical trial has been registered in Chinese Clinical Trial Register, number ChiCTR-INR-17011949, and followed the Consolidated Standards of Reporting Trials Extension (CONSORT Extension) reporting guideline strictly. The trial protocol was conducted according to the Declaration of Helsinki and Good Clinical Practice guidelines, with the approval of ethics committee of Longhua Hospital with the ethic code 2017LCSY326, and subsequently by the relevant ethics committees at all sites. All participants gave their informed consent for inclusion before they participated in the study.

### Participants

#### Inclusion Criteria

Participants were enrolled from June 2017 to November 2018. In this trial, participants were eligible if they were older than 30 years and diagnosed as idiopathic PD [according to the Movement Disorder Society (MDS) Clinical Diagnostic Criteria] at Hoehn and Yahr stages 1–3 (Hoehn and Yahr, 2001; Postuma et al., 2015). Use of levodopa and concomitant anti-parkinsonian medications such as anticholinergic drugs, MAO-B inhibitors, amantadine, catechol-O-methyltransferase inhibitors, or dopamine agonists was allowed if dosages were stable for at least 30 days before enrollment. Moreover, the dose of dopaminergic drugs was allowed to be appropriately adjusted with the approval of neurologists according to the situation of each patient during the trial.

#### Exclusion criteria

Exclusion criteria were receiving treatments for psychiatric disorders; chronic diseases other than PD that could impede full participation in the trial; use of reserpine, metoclopramide, α-methyldopa, amphetamine derivatives, or methylphenidate within the past 3 months; and cognitive impairment (assessed by Abbreviated Mental Test Score <6) (Piotrowicz et al., 2019). We also excluded those who were participating in other clinical trials, or the women who were pregnant or lactating.

### Recruitment and Randomization

We adopted the methods of our previous trial of PCG (Gu et al., 2021). Participants were initially evaluated during a screening visit at which demographic information and eligibility criteria were verified and informed consent was also obtained. Baseline visit occurred within 1 week of the screening visit. At this visit, eligible participants were enrolled and randomly allocated at a 1:1 ratio to PCG or placebo groups. Randomization was performed by random permuted blocks of sizes four to provide a balanced distribution of treatment groups. In order to preserve masking, the trial randomization sequence was generated by an independent study coordinator, with the details of the group assignment concealed on cards placed inside sequentially numbered, opaque sealed envelopes. Drugs were allocated to participants by interviewers. PCG and placebo were prepared and packaged by Jiangyin Tianjiang Pharmaceutical Co., Ltd., of China, which were identical and could not be differentiated. Blind methods were applied for both participants and researchers including interviewers and assessors of the outcomes to ensure the authenticity of statistical results. Blindness was revealed only after all the data were collected or serious adverse events occurred with the approval of the steering committee.

### Interventions

Both PCG group and placebo group received the double-blind maintenance treatment lasting 24 weeks (PCG solution with 8 g of PCG dissolving in 200 ml water twice per day, or matching placebo). PCG (8 g per bag) was made from modern techniques of extraction, concentration, dryness, granulation, and packaging. The matching placebo contained 10% PCG, as well as edible lactose, bitterant, starch, and pigment. Concomitant anti-parkinsonian medications were kept, and dosages could be changed in case of newly emergent or worsened dyskinesias throughout the trial. Subjects not taking the prescribed trial drugs for more than 4 days consecutively or using less than 50% of drugs would be defined as having violated the protocol and would not receive subsequent drugs or assessments.

### Outcome Measures

**FIGURE 1:**
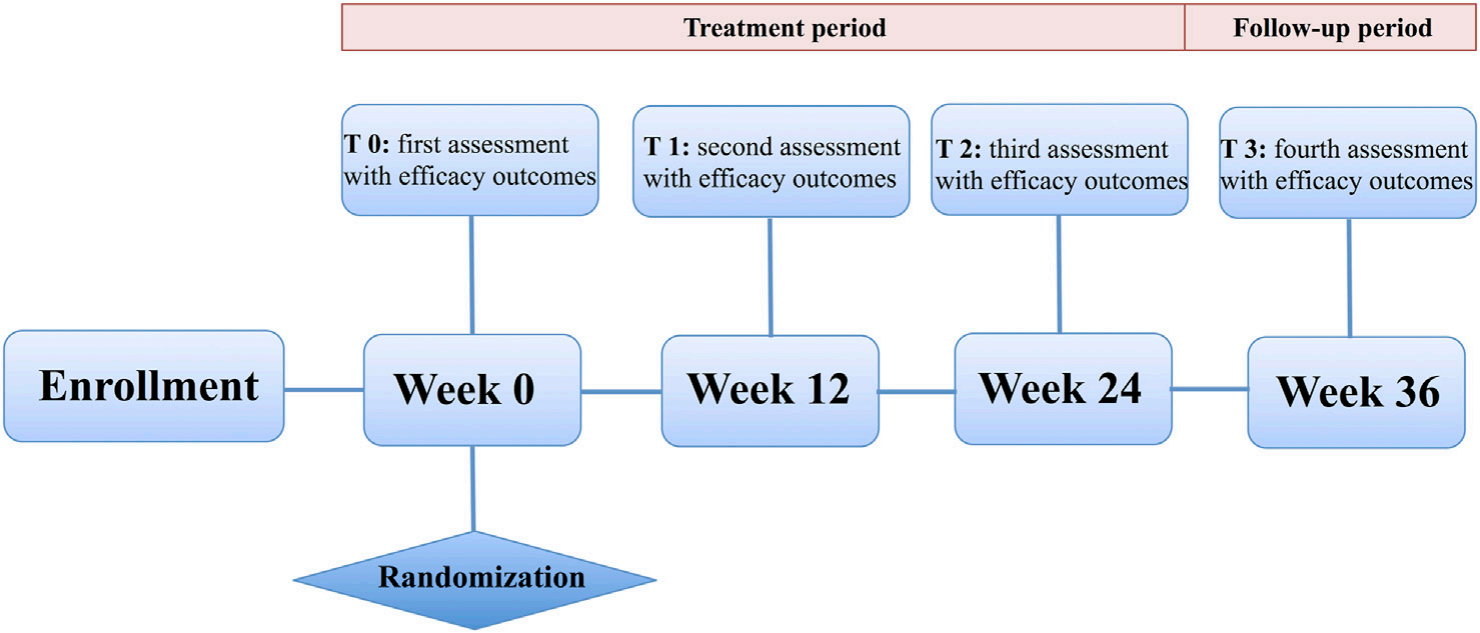
Study design.

Each participant was asked to have a face-to-face clinical assessment at study centers. The assessments of motor function were performed 1 h after taking the first dose of the anti-parkinsonian drugs for participants treated with dopamine replacement therapy at study entry by the same evaluator at all visits for a given patient. As for *de novo* participants, the assessment was conducted 1 h after taking the first dose of the trial drugs. All outcome measures were administered during treatment and follow-up at each time point: baseline/randomization (T0), 12 weeks immediately after the randomization (T1), 24 weeks immediately after the randomization (T2), and 36 weeks immediately after the randomization (12 weeks after treatment, T3) (Figure 1).

The primary analysis comprised two outcomes. The first primary outcome compared the severity of motor symptoms in PD between the PCG and placebo groups at T1, T2, and T3, measured by Unified Parkinson’s disease Rating Scale (UPDRS) part 3 (UPDRS-III), covering domains related to bradykinesia, tremor, rigidity, and postural instability. This comparison did not only determine whether PCG provide benefits on motor dysfunction in PD but also demonstrate whether the treatment effects observed were still present at the end of the study. The second primary outcome compared estimates of slope of the change in total UPDRS score per week between PCG group and placebo group from T0 to T2. This comparison ascertained whether there was a difference in the rate of disease progression, as reflected by the total UPDRS score between two groups. A promising PD-modifying medication would be expected to play a long-lasting treatment role in motor dysfunction and slow the rate of disease progression, as compared with placebo.

Secondary outcomes further assessed the efficacy of PCG for non-motor symptoms in PD, which included 1) activities of daily living as measured by UPDRS part 2 (UPDRS-II) (Martínez-Martín et al., 1994); 2) autonomic dysfunction as measured by the Scale for Outcomes in Parkinson’s Disease-Autonomic (SCOPA-AUT) (Visser et al., 2004); 3) sleep and nocturnal disability as measured by Parkinson’s disease Sleep Scale (PDSS) (Chaudhuri et al., 2002); 4) depressive symptoms as measured by 24-item Hamilton Rating Scale for Depression (HAM-D) (Schrag et al., 2007); 5) anxiety disorders as measured by Hamilton Rating Scale for Anxiety (HAM-A) (Leentjens et al., 2011); and 6) disease-specific quality of life as measured by 39-item Parkinson’s Disease Questionnaire (PDQ-39) regarding mobility, emotional wellbeing, social support, cognition, communication, bodily discomfort, and stigma (Peto et al., 1998) at T1, T2, and T3.

### Adverse Events

Adverse events were identified by questions about significant harm or discomfort caused by the study medications at each visit. Severe adverse events were defined as affecting work or daily activities, with serious adverse events defined as fatal or resulting in disability. Patients were asked to inform researchers if encountering any adverse events related to the trial.

### Sample Size

The estimated difference of UPDRS-III score from baseline to week 24 between treatment groups was set as at least 3.9 points, according to the pilot power calculation based on data from a separate sample of PD patients in our previous study [standard deviation (SD) = 3.9] (Ye et al., 2018). Considering a 5% significance level, the Z value was based on the Z value table for a two-tailed distribution of 1.96. The β value was determined at 0.01, and the Z value based on the Z value table for one tailed distribution of 2.58. The sample size by normal approximation can be determined by the formula as follows (Chow et al., 2008):

$$n_{1}=n_{2}=\frac{2{\left(Z_{\alpha /2}+Z_{\beta }\right)}^{2}\sigma^{2}}{\delta^{2}}=\;\frac{2\;\times \;{\left(1.96+2.58\right)}^{2}\;\times \;6.7^{\text{2}}}{3.9^{\text{2}}}\;\approx 122$$

where n is the sample size of each group, Zα is the table of Z values (two-tailed distribution), Zβ is the table of Z values (one-tailed distribution), σ  is the SD, and δ is the difference. For providing a 2-arm trial with 99% power to detect a difference of UPDRS-III score between groups at least 3.9 at a 2-sided significance level of 5%, a sample size of 292 of the trial population with 146 participants per arm was required with consideration of attrition rate.

### Statistical Analysis

Available data from all randomized participants were included in the analysis, in accordance with the intention-to-treat principle. We used the last-observation-carried-forward strategy for imputation of missing data in statistical analysis.

Descriptive statistics were performed for demographics information and trial outcomes at each time point. The Shapiro–Wilk statistic was used to test the normality of the distribution of all variables. Continuous data were presented as median (interquartile range [IQR]) or mean (SD), with categorical data presented as proportion and number as appropriate. Comparisons were analyzed with use of Student’s t tests or Wilcoxon’s rank-sum tests for continuous data and chi-square tests or Fisher’s exact tests for categorical data. Statistical tests were 2-tailed with a 5% level of statistical significance in this study.

Generalized estimating equation (GEE) models with the first-order autoregressive structure were applied to measure the differential changes in motor and non-motor symptoms in primary and secondary outcomes between PCG and placebo groups. Factors included in the model were treatment group, time, and an interaction of treatment group and time with adjustment for variables of clinical interest [baseline LED, Hoehn and Yahr stage, and motor subtype according to Schiess ratio (tremor-dominant group, postural instability and gait difficulty group, and indeterminate group)]] (Schiess et al., 2000; Tomlinson et al., 2010).

Various prespecified sensitivity and supportive analyses were used to validate the clinical efficacy of PCG. We examined the occurrence of reduction in dose of dopaminergic medication between the two groups in a multivariable logistic regression model, defined as the individual daily levodopa equivalent dose (LED) of participants with baseline anti-parkinsonian therapy at T3 less than T0. Here, we also applied the gradient boosting regression tree (GBDT) to rank the importance of variables with respect to their correlation of baseline LED. GBDT is an ensemble machine learning algorithm combining weak “learners” into a strong single learner in an iteration fashion, which is widely used for both classification and regression problems (Friedman, 2001). In addition, to address the possibility that an effect on symptoms might mask a potential PD-modifying effect in participants with mild disease, a *post hoc* subgroup analysis of the primary outcomes were conducted in the participants with the highest quartiles of UPDRS III and total UPDRS scores at baseline. All statistical analyses in the current study were conducted with R software (version 3.3.3).

## Results

### Baseline Questionnaire and Demographic Information

**FIGURE 2:**
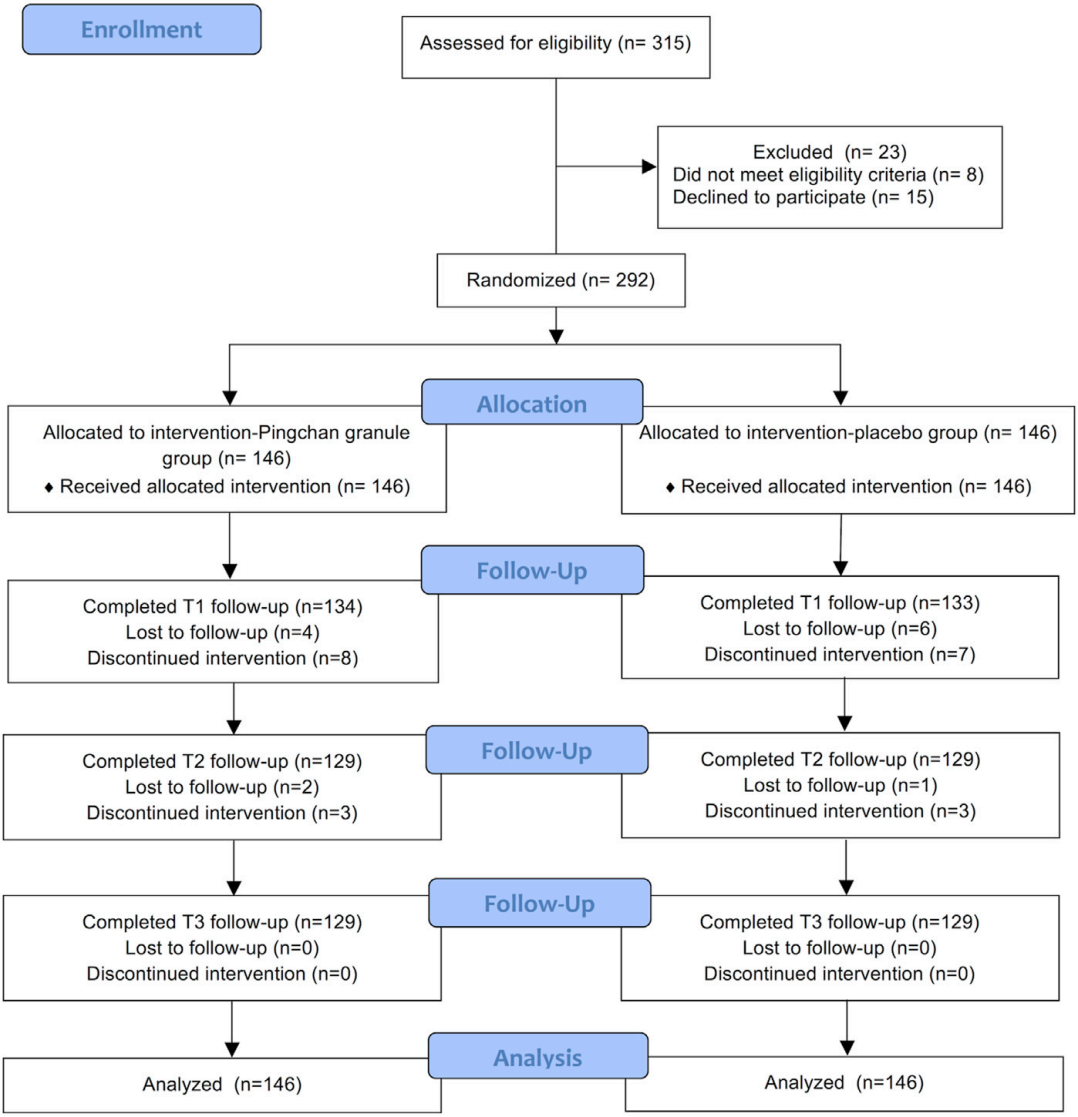
CONSORT diagram. CONSORT, Consolidated Standards of Reporting Trials.

Between June 13, 2017, and November 18, 2018, 315 patients were assessed for study eligibility, of whom 292 were assigned randomly to receive either PCG (*n* = 146) or placebo (*n* = 146). A total of 34 (11.64%) participants dropped out during the trial: 17 (11.64%) in the PCG group and 17 (11.64%) in the placebo group. Detailed information about trial procedures is presented in Figure 2. There was no significant difference between PCG and the placebo group in clinical and demographic characteristics at baseline (Table 2).

**TABLE 2:** Baseline characteristics.

Characteristics	Placebo (*n* = 146)	Pingchan granule (*n* = 146)	*p* value
Age [median (IQR)]]	67.00 (8.00)	67.00 (6.75)	0.65
Age at onset [median (IQR)]	61.50 (8.00)	62.50 (9.00)	0.46
Gender female, n (%)	64 (43.83)	67 (45.89)	0.81
Education [years, median (IQR)]	11.00 (2.00)	11.00 (2.00)	0.72
Duration of PD [years, median (IQR)]	3.90 (5.50)	3.99 (4.46)	0.90
Hoehn and Yahr stage, n (%)			0.46
Stage 1	28 (19.17)	25 (17.12)	
Stage 1.5	52 (35.63)	41 (28.08)	
Stage 2	34 (23.28)	46 (31.51)	
Stage 2.5	20 (13.70)	19 (13.02)	
Stage 3	12 (8.22)	15 (10.27)	
With use of levodopa, n (%)	125 (85.62)	136 (93.15)	0.06
LED [mg/d, median (IQR)]	375.00 (343.75)	375.00 (200.00)	0.83
UPDRS II score [median (IQR)]	10.00 (5.00)	9.00 (7.00)	0.72
UPDRS III score (median (IQR)]	12.00 (8.00)	13.00 (8.00)	0.42
UPDRS total score [median (IQR)]	27.00 (14.00)	26.00 (14.00)	0.93
Motor subtype, n (%)			0.59
Postural instability and gait difficulty	109 (74.66)	112 (76.71)	
Tremor-dominant	21 (14.38)	23 (15.75)	
Indeterminate	16 (10.96)	11 (7.54)	
SCOPA-AUT score [median (IQR)]	8.00 (7.50)	7.00 (8.00)	0.89
PDSS score (median (IQR)]	119.00 (29.00)	116.00 (31.00)	0.88
HAM-D score [median (IQR)]	32.00 (8.50)	31.00 (7.00)	0.15
HAM-A score [median (IQR)]	9.00 (10.00)	8.00 (8.00)	0.17
PDQ-39 score [median (IQR)]	22.00 (30.00)	25.00 (27.00)	0.82

Abbreviations: PD, Parkinson’s disease; LED, levodopa equivalent doses; UPDRS, Unified Parkinson’s disease Rating Scale; SCOPA-AUT, Scale for Outcomes in Parkinson’s Disease–Autonomic; PDSS, Parkinson’s disease Sleep Scale; HAM-D, Hamilton Rating Scale for Depression; HAM-A, Hamilton Rating Scale for Anxiety; PDQ-39, Parkinson’s Disease Questionnaire; IQR, interquartile range.

### Primary Outcomes

**FIGURE 3:**
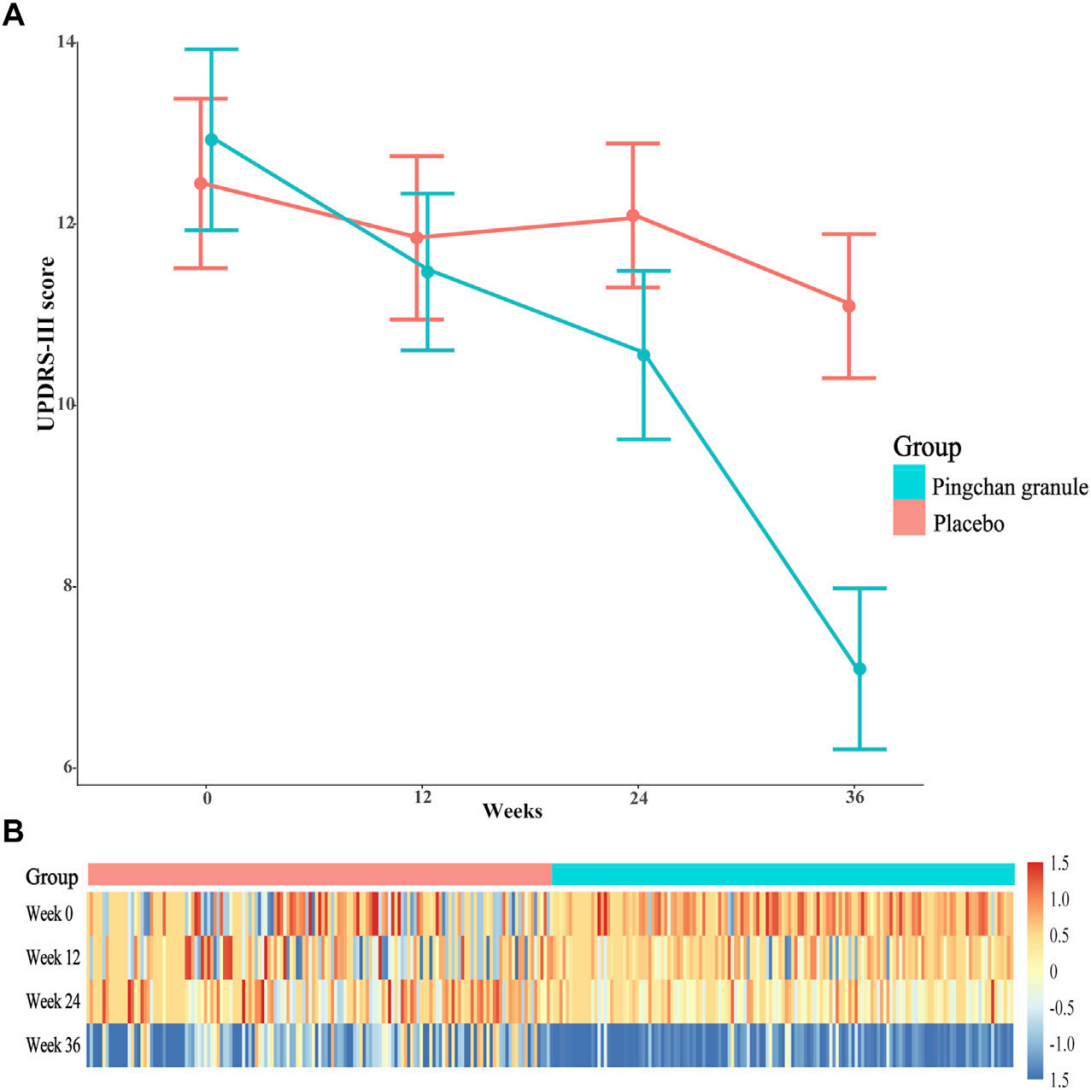
UPDRS-III score during the study by treatment group. **(A)** Data were observed values of UPDRS-III score at T0, T1, T2, and T3 time point. Error bars represented 95% CIs. **(B)** A heat-map of UPDRS-III score for PCG and placebo groups across the 4 time points. Data were log2-transformed with normalization; red implies increased expression while blue implies decreased expression. Abbreviations: UPDRS-III, Parkinson’s disease Rating Scale (UPDRS) part 3; PCG, Pingchan granule; CI, confidence interval; T0, baseline; T1, 12 weeks after the randomization; T2, 24 weeks after the randomization; T3, 36 weeks after the randomization (12 weeks after treatment).

For the first primary outcome comparing the severity of motor symptoms (measured by UPDRS III score) between PCG and placebo at each time point, the groups differed at T1 (*p* = 0.01), T2 (*p* < 0.001), and T3 (*p* < 0.001), as shown in Table 3. The PCG group showed a significant reduction in UPDRS-III score [PCG, T1: β, −1.48 (95% CI, –2.00––0.96, *p* < 0.001); T2: β, −2.40 (95% CI, −2.99–−1.81; *p* < 0.001); T3: β, −5.86 (95% CI, −6.47–−5.25; *p* < 0.001)]; Figure 3) and demonstrated significantly better improvement in UPDRS-III motor score compared with the placebo group [time-by-group interaction, T1: β, −0.92 (95% CI, −1.59–−0.25; *p* = 0.01); T2: β, −2.08 (95% CI, −2.90–−1.27; *p* < 0.001); T3: β, −4.54 (95% CI, −5.37–−3.71; *p* < 0.001)] at T1, T2, and T3 (Table 3; Figure 3). No significant improvement was achieved in motor function across time points in the placebo group.

**TABLE 3:** Generalized estimating equation analysis for the comparison of efficacy outcomes^a^.

Outcome	Median (IQR)			Group effect^b^			Time effect^c^			Group × time effect^d^	
	Pingchan granule	Placebo		β (95% CI)	*p* value		β (95% CI)	*p* value		β (95% CI)	*p* value
UPDRS III score									
T0	13.00 (8.00)	12.00 (8.00)		0.06 (−0.93–1.09)	0.88		NA	NA		NA	NA
T1	11.00 (6.75)	12.00 (7.00)					−0.56 (−0.98 to −0.14)	0.01		−0.92 (−1.59 to −0.25)	0.01
T2	10.00 (7.00)	12.00 (6.00)					−0.32 (−0.87 to 0.24)	0.27		−2.08 (−2.90 to −1.27)	<0.001
T3	6.50 (7.00)	11.00 (6.00)					−1.31 (−2.68 to 0.06)	0.14		−4.54 (−5.37 to −3.71)	<0.001
UPDRS II score									
T0	9.00 (7.00)	10.00 (5.00)		−0.70 (−1.71 to 0.32)	0.18		NA	NA		NA	NA
T1	8.00 (6.00)	9.00 (5.00)					−0.27 (−0.6 to 0.06)	0.11		−0.66 (−1.13 to −0.18)	0.01
T2	8.00 (7.00)	10.00 (5.00)					0.16 (−0.35–0.67)	0.53		−1.60 (−2.27 to −0.94)	<0.001
T3	5.00 (6.00)	9.00 (5.00)					−0.84 (−2.02 to 0.34)	0.21		−3.42 (−4.10 to −2.74)	<0.001
SCOPA-AUT score									
T0	7.00 (8.00)	8.00 (7.50)		0.09 (−1.27–1.45)	0.90		NA	NA		NA	NA
T1	5.00 (6.00)	7.00 (8.00)					−0.93 (−1.55 to −0.3)	0.003		−1.17 (−1.96 to −0.38)	0.004
T2	4.00 (5.00)	6.00 (6.00)					−1.34 (−2.18 to −0.49)	0.002		−1.86 (−2.92 to −0.81)	<0.001
T3	1.00 (5.00)	6.00 (7.00)					−1.64 (−2.49 to −0.79)	<0.001		−4.10 (−5.16 to −3.03)	<0.001
PDSS score									
T0	116.00 (31.00)	119.00 (29.00)		0.02 (−4.85–4.89)	0.99		NA	NA		NA	NA
T1	124.00 (23.00)	120.00 (29.00)					2.22 (0.50–3.95)	0.01		2.99 (0.71–5.27)	0.01
T2	127.00 (18.00)	121.00 (29.00)					3.22 (0.70–5.73)	0.01		6.29 (3.11–9.47)	<0.001
T3	132.00 (17.00)	122.00 (29.00)					4.25 (1.74–6.76)	<0.001		11.78 (8.62–14.95)	<0.001
HAM-D score									
T0	31.00 (7.00)	32.00 (8.50)		−1.41 (−2.85 to 0.03)	0.05		NA	NA		NA	NA
T1	29.00 (6.00)	31.00 (10.00)					−1.35 (−2.19 to −0.50)	0.002		−0.66 (−1.63 to 0.32)	0.19
T2	28.00 (4.00)	31.00 (8.00)					−1.96 (−3.06 to −0.87)	<0.001		−1.16 (−2.43 to 0.10)	0.07
T3	26.00 (4.00)	30.00 (8.00)					−2.49 (−3.59 to −1.39)	<0.001		−2.78 (−4.06 to −1.5)	<0.001
HAM-A score									
T0	8.00 (8.00)	9.00 (10.00)		−1.29 (−2.78 to 0.10)	0.09		NA	NA		NA	NA
T1	6.00 (7.00)	8.00 (10.00)					−1.33 (−1.95 to −0.70)	<0.001		−0.12 (−0.87 to 0.63)	0.08
T2	5.00 (6.75)	8.00 (9.00)					−1.51 (−2.34 to −0.68)	<0.001		−0.92 (−1.92 to 0.07)	0.07
T3	4.00 (6.00)	8.00 (9.00)					−1.51 (−2.34 to −0.68)	<0.001		−2.22 (−3.22 to −1.22)	<0.001
PDQ-39 score									
T0	25.00 (27.00)	22.00 (30.00)		−0.51 (−5.16 to 4.15)	0.83		NA	NA		NA	NA
T1	18.00 (26.80)	21.50 (32.00)					−0.89 (−2.37 to 0.59)	0.24		−2.56 (−4.42 to −0.70)	0.007
T2	16.00 (23.80)	22.00 (28.80)					−1.26 (−3.16 to 0.65)	0.20		−5.14 (−7.75 to −2.52)	<0.001
T3	10.50 (23.80)	16.00 (29.80)					−1.25 (−3.16 to 0.65)	0.19		−10.47 (−13.08 to −7.85)	<0.001

Abbreviations: UPDRS, Unified Parkinson’s disease Rating Scale; SCOPA-AUT, Scale for Outcomes in Parkinson’s Disease–Autonomic; PDSS, Parkinson’s disease Sleep Scale; HAM-D, Hamilton Rating Scale for Depression; HAM-A, Hamilton Rating Scale for Anxiety; PDQ-39, 39-item Parkinson’s Disease Questionnaire; IQR, interquartile range; CI, confidence interval; T0, baseline; T1, 12 weeks after the randomization; T2, 24 weeks after the randomization; T3, 36 weeks after the randomization (12 weeks after treatment).

a The placebo group and the baseline measurement (T0) were the reference categories in the generalized estimating equation model and its corresponding null variables.

b Group effect was defined as group differences at baseline between intervention and control groups.

c Time effect at T1 defined as change of scores for the control group at T1 compared with T0; T2 defined as change of scores for the control group at T2 compared with T0; T3 defined as change of scores for control group at T3 compared with T0.

d Group × time effect at T1 defined as additional change of scores for Pingchan granule group compared with place group at T1; T2 defined as additional change of scores for Pingchan granule group compared with place group at T2; T3 defined as additional change of scores for Pingchan granule group compared with place group at T3.

For the second primary outcome comparing the rate of disease progression between PCG and placebo, the estimated rate of change in total UPDRS score per week from T0 to T2 showed a more rapid rate of improvement tendency (decrease in the total UPDRS score) for PCG [−2.23 (95% CI, −2.72–−1.73; *p* < 0.001) points per week] than for placebo [−0.21 (95% CI, −0.80–0.39; *p* = 0.50) points per week] (*p* < 0.001).

### Secondary Outcomes

**FIGURE 4:**
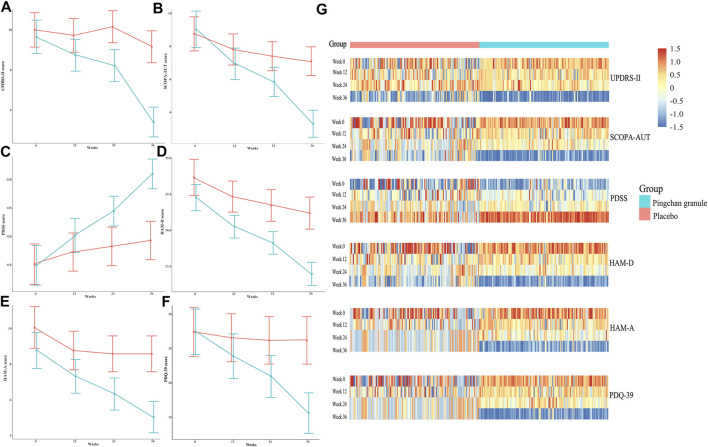
UPDRS-II, SCOPA-AUT, PDSS, HAM-D, HAM-A, and PDQ-39 scores during the study by treatment group. **(A)** Data were observed values of UPDRS-II score at the T0, T1, T2, and T3 time points. **(B)** Data were observed values of SCOPA-AUT score at the T0, T1, T2, and T3 time points. **(C)** Data were observed values of PDSS score at the T0, T1, T2, and T3 time points. **(D)** Data were observed values of HAM-D score at T0, T1, T2, and T3 time point. **(E)** Data were observed values of HAM-A score at T0, T1, T2 and T3 time point. **(F)** Data were observed values of HAM-D score at the T0, T1, T2, and T3 time points. Error bars represented 95% CIs. **(G)** Heat-maps of UPDRS-II, SCOPA-AUT, PDSS, HAM-D, HAM-A, and PDQ-39 scores for PCG and placebo groups across the 4 time points. Data were log2-transformed with normalization; red implies increased expression while blue implies decreased expression. Abbreviations: UPDRS-II, Parkinson’s disease Rating Scale (UPDRS) part 2; SCOPA-AUT, Scale for Outcomes in Parkinson’s Disease–Autonomic; PDSS, Parkinson’s disease Sleep Scale; HAM-D, Hamilton Rating Scale for Depression; HAM-A, Hamilton Rating Scale for Anxiety; PDQ-39, 39-item Parkinson’s Disease Questionnaire; PCG, Pingchan granule; CI, confidence interval; T0, baseline; T1, 12 weeks after the randomization; T2, 24 weeks after the randomization; T3, 36 weeks after the randomization (12 weeks after treatment).

The PCG group showed significant improvements in UPDRS-II activities of daily living score [T1: β, −0.93 (95% CI, –1.27––0.58, *p* < 0.001); T2: β, −1.44 (95% CI, −1.87–−1.01; *p* < 0.001); T3: β, −4.26 (95% CI, −4.70–−3.81; *p* < 0.001)], SCOPA-AUT autonomic score [T1: β, −2.10 (95% CI, –2.58––1.61, *p* < 0.001); T2: β, −3.20 (95% CI, −3.84–−2.57; *p* < 0.001); T3: β, −5.73 (95% CI, −6.38–−5.09; *p* < 0.001)], PDSS sleep disability score [T1: β, 5.21 (95% CI, 3.73–6.70, *p* < 0.001); T2: β, 9.51 (95% CI, 7.57–11.45; *p* < 0.001); T3: β, 16.04 (95% CI, 14.11–17.96; *p* < 0.001)], HAM-D depression score (T1: β, −2.00 (95% CI, –2.50––1.51, *p* < 0.001); T2: β, −3.13 (95% CI, −3.77–−2.49; *p* < 0.001); T3: β, −5.27 (95% CI, −5.92–−4.62; *p* < 0.001)], HAM-A anxiety score [T1: β, −1.44 (95% CI, –1.86––1.03, *p* < 0.001); T2: β, −2.44 (95% CI, −2.99–−1.89; *p* < 0.001); T3: β, −3.73 (95% CI, −4.29–−3.17; *p* < 0.001)], and PDQ-39 disease-specific quality of life score [T1: β, −3.45 (95% CI, –4.58––2.32, *p* < 0.001); T2: β, −6.39 (95% CI, −8.18–−4.60; *p* < 0.001); T3: β, −11.72 (95% CI, −13.50–−9.94; *p* < 0.001)] across all time points (Table 3; Figure 4).

In the placebo group, significant improvements were noted in SCOPA-AUT score [T1: β, −0.93 (95% CI, −1.55–−0.30, *p* = 0.003); T2: β, −1.34 (95% CI, −2.18–−0.49; *p* = 0.002); T3: β, −1.64 (95% CI, −2.49–−0.79; *p* < 0.001)], PDSS sleep disability score (T1: β, 2.22 (95% CI, 0.50–3.95, *p* = 0.01); T2: β, 3.22 (95% CI, 0.70–5.73; *p* = 0.01); T3: β, 4.25 (95% CI, 1.74–6.76; *p* < 0.001)], HAM-D depression score [T1: β, −1.35 (95% CI, −2.19–−0.50, *p* = 0.002); T2: β, −1.96 (95% CI, −3.06–−0.87; *p* < 0.00); T3: β, −2.49 (95% CI, −3.59–−1.39; *p* < 0.001)], and HAM-A anxiety score [T1: β, −1.33 (95% CI, −1.95–−0.70, *p* < 0.001); T2: β, −1.51 (95% CI, −2.34–−0.68; *p* < 0.001); T3: β, −1.51 (95% CI, −2.34–−0.68; *p* < 0.001)] at the T1, T2, and T3 time points. No significant improvement was noted in the UPDRS-II and PDQ-39 scores of the placebo group across time points (Table 3; Figure 4).

Compared with the placebo group, the PCG group demonstrated significantly better improvement in UPDRS-II score [time-by-group interaction, T1: β, −0.66 (95% CI, −1.13–−0.18, *p* = 0.01); T2: β, −1.60 (95% CI, −2.27–−0.94; *p* < 0.001); T3: β, −3.42 (95% CI, −4.10–−2.74; *p* < 0.001)], SCOPA-AUT score [time-by-group interaction, T1: β, −1.17 (95% CI, −1.96–−0.38, *p* = 0.004); T2: β, −1.86 (95% CI, −2.92–−0.81; *p* < 0.001); T3: β, −4.10 (95% CI, −5.16–−3.03; *p* < 0.001)], PDSS score [time-by-group interaction, T1: β, 2.99 (95% CI, 0.71–5.27, *p* = 0.01); T2: β, 6.29 (95% CI, 3.11–9.47; *p* < 0.001); T3: β, 11.78 (95% CI, 8.62–14.95; *p* < 0.001)], HAM-D score [time-by-group interaction, T3: β, −2.78 (95% CI, −4.06–−1.5; *p* < 0.001)], HAM-A score [time-by-group interaction, T3: β, −2.22 (95% CI, −3.22 to −1.22; *p* < 0.001)], and PDQ-39 score [time-by-group interaction, T1: β, −2.56 (95% CI, −4.42–−0.70, *p* = 0.007); T2: β, −5.14 (95% CI, −7.75–−2.52; *p* < 0.001); T3: β, −10.47 (95% CI, −13.08–−7.85; *p* < 0.001); Table 3; Figure 4].

### *Post-hoc* analyses

**FIGURE 5:**
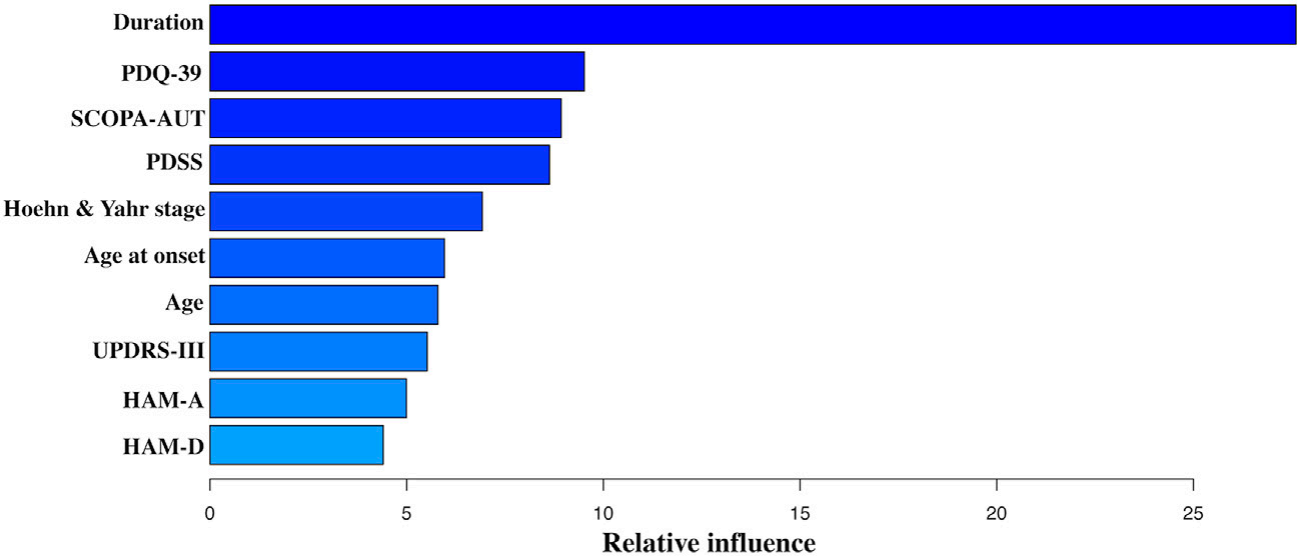
Variable importance derived from the GBDT model. Abbreviations: GBDT, gradient boosting regression tree; UPDRS-III, Parkinson’s disease Rating Scale (UPDRS) part 3; SCOPA-AUT, Scale for Outcomes in Parkinson’s Disease–Autonomic; PDSS, Parkinson’s disease Sleep Scale; HAM-D, Hamilton Rating Scale for Depression; HAM-A, Hamilton Rating Scale for Anxiety; PDQ-39, 39-item Parkinson’s Disease Questionnaire.

The frequency of reduction in dose of dopaminergic drugs at T3 showed a more positive outcome in the PCG group than in the placebo group [38/101 (37.63%) in the PCG group vs. 21/113 (18.58%) in the placebo group; OR = 2.20, 95% CI (1.20, 4.14), *p* = 0.01]. Moreover, there was a modest decrease of LED in patients allocated PCG at T3 [T0: 375.00 (IQR 343.75) mg/day vs. T3: 337.50 (IQR 275.00) mg/day; *p* < 0.001], while there was no significant reduction observed in those assigned placebo. GBDT identified PD duration, baseline Hoehn and Yahr stage, age of PD onset, age, baseline PDQ-39, SCOPA-AUT, PDSS, UPDRS-III, HAM-A, and HAM-D scores as top 10 predictors of baseline LED. Figure 5 reflected the contribution made by each variable in predicting baseline LED.

To address the possibility that PCG had an effect on symptoms that might have masked a potential treatment effect in subjects with a low UPDRS score, the primary analyses were performed for subjects with UPDRS score in the highest quartiles (UPDRS III score >11.2 points and total UPDRS score >23.6 points) at baseline. In the subjects with UPDRS III score >11.2 points (*n* = 538), the PCG group again showed a significant reduction in UPDRS-III score [PCG, T1: β, −1.70 (95% CI, –2.45––0.95, *p* < 0.001); T2: β, −1.75 (95% CI, −2.55–−0.95, *p* < 0.001); T3: β, −4.91 (95% CI, −5.78–−4.04, *p* < 0.001)] and demonstrated significantly better improvement in UPDRS-III motor score compared with the placebo group [time-by-group interaction, T1: β, −1.10 (95% CI, −1.98–−0.21; *p* = 0.01); T2: β, −0.87 (95% CI, −1.93–−0.19; *p* = 0.11); T3: β, −3.11 (95% CI, −4.25–−3.11; *p* < 0.001)]. For the second primary outcome, the estimated rate of change in total UPDRS score per week in the subjects with total UPDRS score >23.6 points (*n* = 564) from T0 to T2 also showed a larger damping of total UPDRS score for PCG [−2.40 (95% CI, −2.94–−1.86; *p* < 0.001) points per week] than for placebo [−0.92 (95% CI, −1.51–−0.33; *p* = 0.002) points per week] (*p* < 0.001).

### Adverse Events

All the reported adverse events during the study are listed in Table 4 and all adverse events were mild and transient. No serious adverse events were reported. There were no significant between-group differences in the occurrence of adverse events. None of the patients discontinued the study due to any adverse events.

**TABLE 4:** Adverse events.

Event	Placebo	Pingchan granule	*p* value
	(*n* = 146)	(*n* = 146)	
Nausea, n (%)	2 (1.37)	2 (1.37)	1.00
Constipation, n (%)	1 (0.85)	2 (1.37)	1.00
Dizziness, n (%)	1 (0.85)	1 (0.85)	1.00
Headache, n (%)	1 (0.85)	1 (0.85)	1.00

## Discussion

This randomized, double-blind, placebo-controlled trial with the cohort of 292 mild-to-moderate PD subjects from multiple centers showed that PCG was superior to the matching placebo for managing motor and non-motor symptoms of PD during the 24-week treatment, which persisted throughout the 12-week follow-up.

### Treatment Effect of PCG on Both Motor and non-motor Symptoms in PD

In the current study, the PCG group had greater improvement in the motor outcomes at T1, T2, and T3 compared with the placebo group, which was further validated in the subgroup of subjects with a high UPDRS III score. To minimize the potential confounding bias, baseline covariates including LED, Hoehn and Yahr stage, and motor subtype were adjusted, supporting the beneficial clinical effect of PCG on motor symptoms.

A growing body of studies identifies a variety of combinations of non-motor symptoms as a key driver of quality of life in PD (Havlikova et al., 2008; Müller et al., 2013; Connolly and Lang, 2014). There were 4-fold main concerns existing in selection of optimal medical treatments for non-motor symptoms in PD. First, most symptomatic treatments used for non-motor symptoms are like treatments for these symptoms in non-PD populations to a certain extent, which are not PD-specific or working *via* dopamine. Droxidopa, probiotics, and sildenafil are possibly useful for orthostatic hypotension, constipation, and sexual dysfunction, while failing to work on motor symptoms (Barboza et al., 2015; The collaborators of the Parkinson’s Disease Update on Non-Motor Symptoms Study Group on behalf of the Movement Disorders Society Evidence-Based Medicine Committee et al., 2019). Second, adverse effect profiles related to combination of anti-parkinsonian drugs or not should not be overlooked. For depression in PD, the risk of combining antidepressants with serotonergic properties and MAO-B inhibitors is the life-threatening serotonin syndrome (Pd Med Collaborative Group et al., 2014; Smith et al., 2015). Similarly, a high dosage of pramipexole is useful for some PD patients with depression but faced with the impulse control disorders (Barone et al., 2010; Garcia-Ruiz et al., 2014). Furthermore, benzodiazepines might help sleep but could worsen cognitive function. Agents with anticholinergic properties may improve sialorrhea or urinary dysfunction but contribute to hallucinations and confusion (Srivanitchapoom et al., 2014; Armstrong and Okun, 2020). Third, the key concern is the likelihood of treatment efficacy and universality, as all the medications mentioned above are only valid for a tiny fraction of non-motor symptoms and high-quality evidence for these treatments specifically in PD patients is not adequate enough. Fourth, dopaminergic therapy might improve certain aspects of non-motor symptoms (such as primary pain and fatigue related to PD), which has to be balanced against the fact that some non-motor symptoms (such as dementia and psychosis in PD) might be exacerbated by dopaminergic drugs (Chaudhuri and Schapira, 2009).

Considering the secondary outcomes, it was remarkable that in this PD-specific study, PCG has established symptomatic treatment efficacy not only in motor symptoms but also in a relatively wide range of non-motor symptoms. The significant treatment differences of 4.10 points, 11.78 points, 2.78 points, 2.22 points, 3.42 points, and 10.47 points in SCOPA-AUT, PDSS, HAM-D, HAM-A, UPDRS-II, and PDQ-39 scores were observed at T3.

Few published trials have prospectively investigated the relatively long-term period impact of PCG on PD. Consequently, we evaluated whether the treatment effect of PCG lasted until 12 weeks after intervention (T3) in this study. Unsurprisingly, we found that the treatment effect of PCG was not only maintained but also enhanced along a 12-week follow-up period, demonstrating that PCG might exert a long-lasting beneficial effect to counter both motor and non-motor symptoms in PD. In summary, the treatment effect of PCG was PD-specific, potent, long-lasting, and extensive, coupled with good tolerability and favorable synergistic effects with the combined anti-parkinsonian therapy, suggesting that PCG was an effective treatment option for PD, as well as a useful adjunct to dopaminergic medications.

The symptomatic efficacy of PCG in PD was supported by laboratory studies. In animal experiments, PCG demonstrated neuroprotective effects in models of PD by inhibiting hyperactivation of c-Jun N-terminal kinase and extracellular signal-regulated kinase (ERK) pathways, and the overexpression of α-synuclein to reduce the apoptosis of neurons and inflammatory reaction of substantia nigra cells, as well as antagonizing the process of misfolded α-synuclein aggregation (Ye et al., 2014b; Ye et al., 2016b; Ye et al., 2017).

### Possible Disease Modification by PCG

It was valuable that this study provided an opportunity to compare estimates of slope of the change in total UPDRS points per week between PCG and placebo groups, determining whether there was a difference in the rate of disease progression. There was a greater improvement in the rate of change in the total UPDRS score between T0 and T2 as compared with placebo, which was again validated by the subgroup analysis in the subjects with a high total UPDRS score.

As PD progresses, more frequent and higher LEDs are required mainly for the inability to store excess dopamine and decreasing erratic responses to dopaminergic medication, which is associated with the emergence of levodopa-induced dyskinesia (LID), in particular after several years of prolonged levodopa treatment (Ahlskog and Muenter, 2001; Scott et al., 2016). Consequently, LED was identified as a main risk factor for disease advancing, as well as a potential marker of PD progression in previous studies on PD (Cucca et al., 2015; Kasamo et al., 2019; Hommel et al., 2020). Furthermore, to our knowledge, this was the first study to investigate clinical determinants of LED. The most powerful predictor of LED was PD duration. Except for 4 PD-specific and demographic variables (PD duration, baseline Hoehn and Yahr stage, age of PD onset, and age), it should also be nominated that one motor parameter (baseline UPDRS-III score) and five non-motor parameters (baseline PDQ-39, SCOPA-AUT, PDSS, HAM-A, and HAM-D scores) contributed to LED. This finding did not only evaluate the dopaminergic response of both motor and non-motor symptoms but also verify the marker value of LED for PD progression.

The rate of decline in LED was also assessed in the current study, and subjects in the PCG group were more likely to reduce LED than the placebo group. Although the drop of LED in the PCG group was modest at T3, it was noteworthy that PCG tended to allow the reduction of the need for a progressive rise in dopaminergic therapy at the early and middle stages of PD. Our results provided insights into the possible disease modification by PCG at the early or middle stage of PD. However, it should be noted that there was no significant change in LED in the placebo group, which might be caused by the relatively short observation time and low-dose use of PCG. Thus, in future studies on PCG, it would be important to apply the delayed-start design and include subjects with more advanced disease in a longer follow-up period for verifying this PD-modifying effect.

### Placebo Effects in PD

PD has been identified as one of neurology disorders for which the rates of placebo response are high. The placebo effects in PD are likely to be mediated by activation of the entire dopaminergic system (Quattrone et al., 2018). In this study, reductions of SCOPA-AUT, PDSS, HAM-D, and HAM-A scores in the placebo group were also detected. These results partially confirmed the hypothesis that patients showed placebo responses characterized by improvements in non-motor symptoms (Diederich and Goetz, 2008), but there was inconsistent evidence of placebo-induced clinical benefits in motor symptoms, as the UPDRS motor section in the placebo group did not differ substantially from T0 to T3 in the present study. To separately evaluate the contributions of placebo factors on active interventions, the assessment of mediators of placebo effects including perceived group assignment and expected benefits would be conducted in future studies (Price et al., 2008).

### Safety

The proportions of adverse events in the PCG and placebo groups in this study were low, and the adverse events were mild or transient, confirming the safety of PD.

### Strengths, Limitations, and Directions for Future Research

The strengths included a double-blinded, multicenter randomized clinical design with adequate statistical power and relatively large sample size to detect a clinically meaningful effect, various follow-up time points to demonstrate the residual effects of interventions, and consistency across results of *post-hoc* analyses.

Limitations should be acknowledged. One limitation was that the majority (89.38%) of participants were taking levodopa at study entry, indicating the inclusion of a minority of *de novo* patients, which might produce some heterogeneity in the observed treatment response. Another limitation was that outcomes regarding autonomic symptoms, sleep disturbances, and psychological disorders in the present study were subjective self-reported, which might induce evaluation bias. In the future, functional and histological evaluations such as quantitative thermal sensory testing (QST), sympathetic skin response (SSR), nerve conduction velocity (NCV), and polysomnography would be applied in our trials on PCG (Ludwig et al., 2007).

## Conclusion

In conclusion, the current study targeting motor and non-motor symptoms as treatment indications investigated and extended the efficacy and safety of PCG examined in the pilot studies to a broader patient population and a more pragmatic setting in the short- and long-term periods. This trial provided level 1 evidence that PCG alleviated motor symptoms and non-motor symptoms in PD, showing that PCG was a valuable alternative therapeutic option for the management of PD. Further research of PCG with delayed-start study design and wider enrollment of patients is warranted.

## Data Availability

The raw data supporting the conclusion of this article will be made available by the authors, without undue reservation.
