# Supplementary material for: Pingchan Granule for Motor Symptoms and Non-Motor Symptoms of Parkinson’s Disease: A Randomized, Double-Blind, Placebo-Controlled Study
Source: Front Pharmacol. 2022 Feb 25;13:739194. doi: 10.3389/fphar.2022.739194 (PMC8914044; doi:10.3389/fphar.2022.739194)
Supplement: Supplementary file 1 [file DataSheet1.DOCX]

**Supplementary Material**

Component identification of Pingchan granule


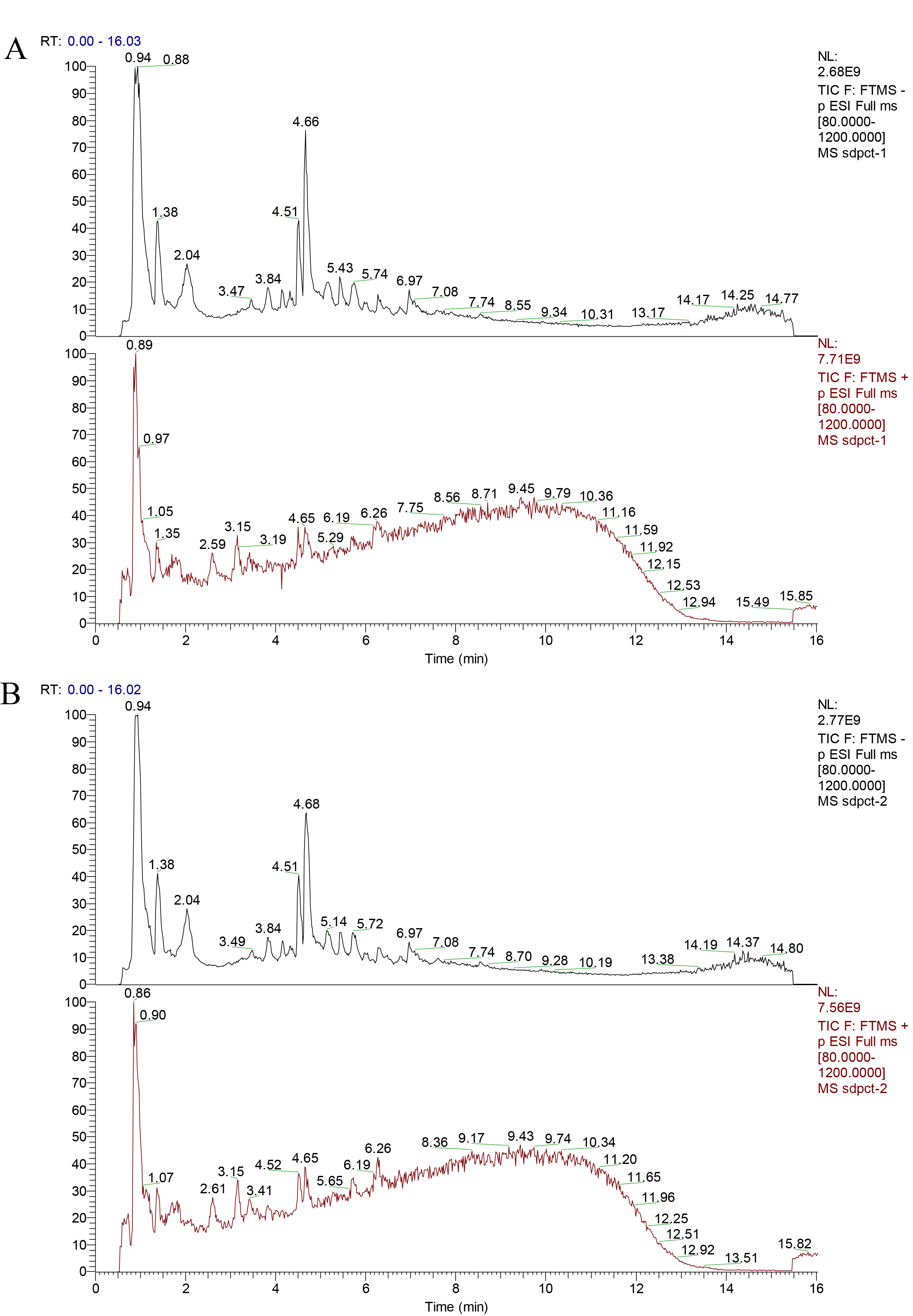


Supplementary figure 1. Schematic diagram of total ion chromatography (TIC) of Pingchan granule. (A) Sample 1 of Pingchan granule. (B) Sample 2 of Pingchan granule.


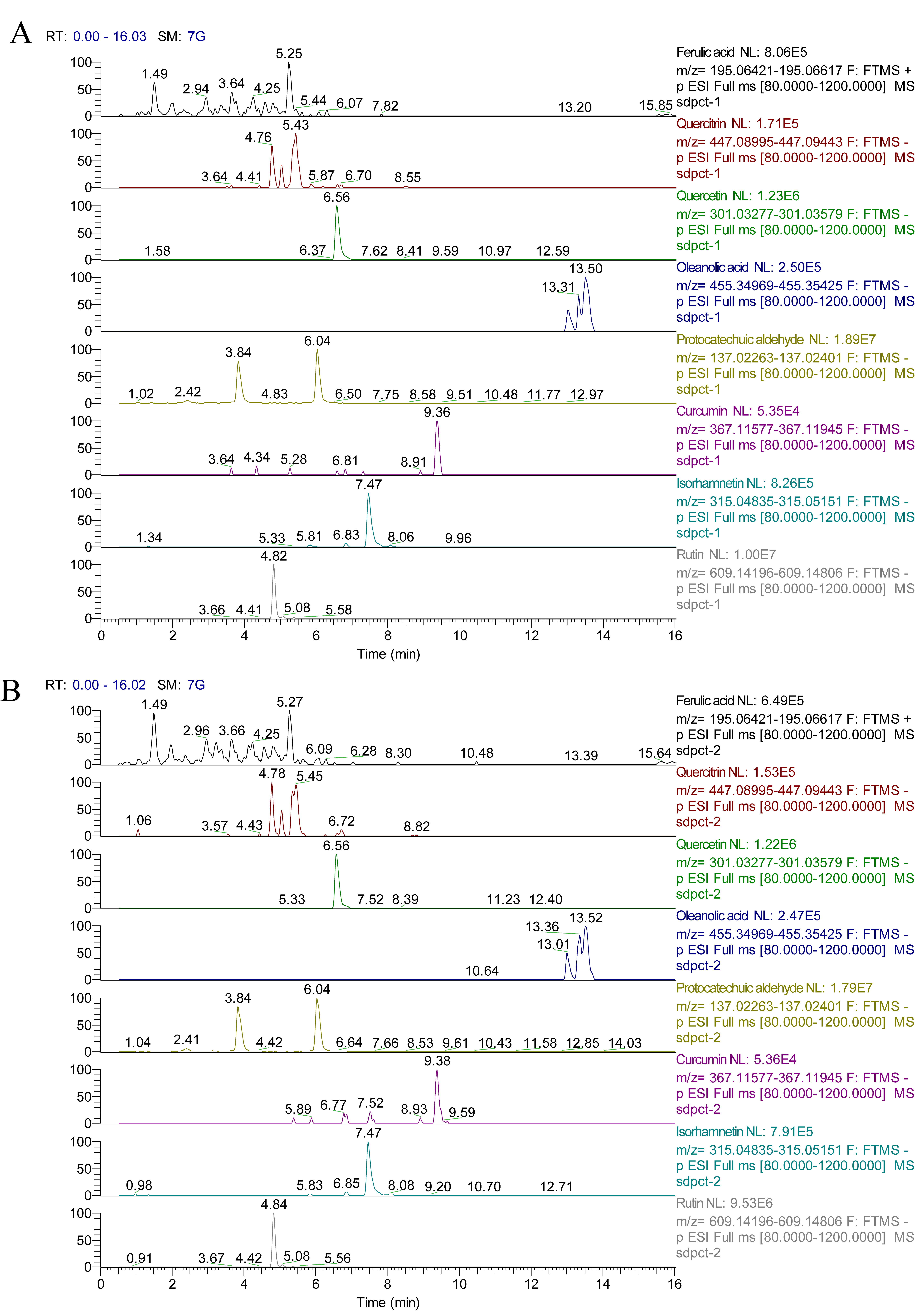


Supplementary figure 2. Single ion monitoring (SIM) chromatogram of Pingchan granule. (A) Sample 1 of Pingchan granule. (B) Sample 2 of Pingchan granule.

Supplementary table 1. Eight compounds of Pingchan granule

| Chemical determination | Content |
| --- | --- |
| Ferulic acid | 20.052 μg/ g |
| Quercitrin | 0.686 μg/ g |
| Quercetin | 1.089 μg/ g |
| Oleanolic acid | 467.907 μg/ g |
| Protocatechuic aldehyde | 6.157 μg/ g |
| Curcumin | 0.070 μg/ g |
| Isorhamnetin | 0.890 μg/ g |
| Rutin | 70.442 μg/ g |

Reference

1. Liu J, Chen L, Fan C, et al. Qualitative and quantitative analysis of major constituents of Paeoniae Radix Alba and Paeoniae Radix Rubra by HPLC-DAD-Q-TOF-MS/MS. China journal of Chinese Material Medica, 2015, 40(9):1762-1770.

2. He H, Ma S, Tian S, et al. HPLC determination of six components in zedoary turmeric oil and its related injections. China journal of Chinese Material Medica, 2010, 35(5):593-597.

3. Bai G, Li Y, Zhang Z, et al. Establishment of HPLC fingerprint of Lycium barbarum and determination of three components. Chinese Traditional Patent Medicine, 2019, 41(7): 1721-1724.

4. Wang J, Zhang M, Zhang X. Simultaneous determination of quercetin and kaempferol in Bombyx Batryticatus by RP-HPLC. Feed Industry. 2009, 30(12): 48-50.

5. Tian X, Fu T, Guo L, et al. HPLC simultaneous determination of five nucleoside compounds in extracts of buthus martensii by different extraction technologies. Chinese Journal of Experimental Traditional Medical Formulae. 2013, 19(8): 13-16.

6. Lu S, Gan Y, Tang L, et al. Evaluation of the Quality of Taxillus chiueusis from Different Hosts Sources by HPLC Fingerprint Combined with Chemometrics. China Pharmacy. 2020, 31(7): 794-799.

7. Shan M, Zhang L, Yu S, et al. Simultaneous determination of eight active components in Gastrodiae Rhizoma by HPLC-MS. Chinese Traditional and Herbal Drugs. 2015, 46(14): 2087-2091.

8. Yang Z, Wang F, Jin C, et al. Comparative study on the HPLC characteristic chromatogram of water extracts of Arisaematis Rhizoma and its processed products. Chinese Traditional and Herbal Drugs. 2020, 51(3): 639-2091.

9. Fu Y, Li Z, Pu S, et al. Chemical constituents from Scolopendra multidens. Chinese Traditional and Herbal Drugs. 2013, 44(13): 1726-1729.
